# Modulation of frequency-tuning property of subcortical neurons elicited by corticofugal signals in bat’s auditory cortex

**DOI:** 10.1186/1471-2202-12-S1-P231

**Published:** 2011-07-18

**Authors:** Yoshitaka Mutoh, Yoshihiro Nagase, Yoshiki Kashimori

**Affiliations:** 1 Department of Engeneering Science, University of Electro-Communications, Chofu, Tokyo 192-8585, Japan; 2 Graduate School of Information Systems, University of Electro-Communications, Chofu Tokyo 182-8585, Japan

## Background

Most species of bat making echolocation use Doppler-shifted frequency of ultrasonic echo pulse to measure the velocity of target [1]. To perform the fine-frequency analysis, the feedback signals from cortex to subcortical and peripheral areas are needed. The feedback signals are known to modulate the tuning property of subcortical neurons. Xia and Suga [2] have shown on an intriguing property of feedback signals that the electric stimulation of cortical neurons evokes the best frequency (BF) shifts of subcortical neurons away from the BF of the stimulated cortical neuron (centrifugal BF shift) and bucuculine (an antagonist of inhibitory GABA receptors) applied to the stimulation site changes the centrifugal BF shifts into the BF shifts towards the BF of stimulated cortical neurons (centripetal BF shift). Although these BF shifts are generated by the feedback signals from cortical neurons to subcortical neurons, it is not yet clear how the feedback signals determine the direction of BF shift. To address this issue, we present a neural model for detecting Doppler-shifted frequency of echo sound reflecting from a target.

## Model

We propose a network model for detecting the Doppler-shifted frequency [3]. The model consists of cochlea (Ch), inferior colliculus (IC), and Doppler-shifted constant frequency (DSCF) area, each of which is a linear array of frequency-tuned neurons. The three layers construct a tonotopical map, in which the neurons in each layer are tuned in to specific echo frequency ranging from 60.0 to 63.0 kHz, corresponding to the frequency range of the second harmonics. The bat uses the Doppler-shifted frequency of echo sound to detect the relative velocity of target. The neurons in the three layers are reciprocally connected with each other, with on center-off surrounding connections. The neurons in different layers are connected with an excitatory and inhibitory synapse, whose weights are updated with a learning with short-term dynamics.

## Results

Our model reproduced well the two types of BF shifts and could explain systematically the neural mechanism underlying these BF shifts. We also showed that the DSCF neurons followed rapidly the frequency change of the moving target. The detection ability of DSCF neurons is due to a fast synaptic change of top-down connections from DSCF to IC neurons.

## Conclusion

We have presented a network model of the Doppler shifted frequency. The model well reproduced the two types of BF shifts observed by Xiao and Suga [2]. The synaptic weights rapidly changed, enabling DSCF neurons to detect the temporal varying signals such as echo signal reflecting from a moving target.
